# Protective Effect of *Tragopogon Graminifolius* DC Against Ethanol Induced Gastric Ulcer

**DOI:** 10.5812/ircmj.7845

**Published:** 2013-09-05

**Authors:** Mohamad Hosein Farzaei, Mozafar Khazaei, Zahra Abbasabadei, Maryam Feyzmahdavi, Gholam Reza Mohseni

**Affiliations:** 1 Fertility and Infertility Research Center, Kermanshah University of Medical Sciences, Kermanshah, IR Iran; 2 Department of Traditional Pharmacy, Faculty of Traditional Medicine, Tehran University of Medical Science, Tehran, IR Iran; 3 Faculty of Pharmacy, Kermanshah University of Medical Sciences, Kermanshah, IR Iran; 4 Akhtar Hospital, Shahid Beheshti University of Medical Sciences, Tehran, IR Iran

**Keywords:** Gastric Ulcer, Protective Effect, Tragopogon, Traditional Medicine

## Abstract

**Background:**

Gastric ulcer is a serious digestive system problem and affects 5% to 10% of people during their life. Chemical antigastric ulcer drugs have side effect, cannot prevent recurrence of ulcer and also show drug interaction with many other medicaments. Tragopogon graminifolius DC.(TG) is a herb which is widely used in the west of Iran and traditionally consumed for the treatment of gastrointestinal disorders. TG was introduced as one of the most beneficial plants for digestive ulcer in Iranian traditional medicine.

**Objectives:**

The aim of the present study was to determine the acute toxicity and protective effect of hydroalcoholic extract of TG (HeTG) against ethanol induced gastric ulcer.

**Materials and Methods:**

Male Wistar rats were divided into five groups (n = 7). HeTG at the doses of 50, 100, and 150 mg/kg were administered orally for 15 days and gastric ulcer was induced by pure ethanol (1 ml/200gr body weight). Ulcer index and protective rate were calculated and histological changes were determined.

**Results:**

HeTG was nontoxic up to 2000 mg/Kg. Ulcer index decreased in extract groups significantly. Protective rates of HeTG were 48.94%, 46.39%, and 43.99% in 50, 100, and 150 mg/kg extract, respectively. 50 mg/kg HeTG group had higher protective effect. There was relatively normal cellular arrangement in HeTG groups.

**Conclusions:**

TG showed protective effect against ethanol induced gastric ulcer. This study confirmed traditional medicine claims of TG.

## 1. Background

Gastric ulcer is the most common digestive problem in clinical examination and affects 5% to 10% of people during their life (1). Gastric ulcer is a complex and multifactorial disease with its incomplete understood etiology. It appears as a pathologic lesion in digestive tract exposed to ulcerogenic agents. This disease was seen as a result of imbalance between invasive and defensive factors (2, 3).

One of the external invasive factors is ethanol which, as other factors like H. pylori infection, nonsteroidal anti-inflammatory drugs (NSAIDs) and steroids, increases gastric ulcer risk. Factors involved in gastric ulcer pathogenesis are free radical production, inhibition of cell proliferations, inflammatory cell infiltrations, and production of reactive oxygen spices from lipid peroxidation and protein oxidation (3).

There are different chemical drugs for gastric ulcer prevention and treatment. Chemical gastric antiulcer drugs such as proton pump inhibitor and H_2_ receptor antagonist have side effects, cannot prevent recurrence of ulcer, and also show drug interaction with many other drugs (4).

Using herbal remedies for prevention and treatment of many diseases are under development worldwide. Numerous researches on medicinal plants have been performed to recognize and classify their application for the prevention and treatment of gastric ulcer. Herbal drugs, especially those that have traditionally been consumed, are safe, clinically effective, and relatively cheaper. They also have comprehensive ability to compete with chemical drugs and are more tolerated by patients during treatment period (1).

Tragopogon graminifolius DC (TG) Known as “sheng” from Compositae (Asteraceae) family is widely consumed as a green vegetable in the west of Iran. In Iranian traditional medicine, TG is used for poison elimination and as astringent and bleeding inhibitor, wound healer, aseptic property, and liver and stomach protector. It is also used for healing digestive bleeding and pulmonary and digestive ulcer. This herb was introduced as one of the most beneficial plants for digestive ulcer in traditional medicine (5, 6). In different nations, Tragopogon genus is used as anticough, astringent, skin repairing (7) and is used in the treatment of gastric disorders traditionally (8).

Active constitutes of Tragopogon genus are flavonoids which consist of apigenin, luteolin, quercetin, vitexin, isovitexin, vicenin-1and 2, swertisin, orientin, isoorientin, and lucenin (9, 10). Some Tragopogon species have triterpene saponins like tragopogonosides A-I, (11) and vitamin C, K and E, (12) were recognized from some Tragopogon species. To our knowledge, there is no scientific report on gastric protective effects of Tragopogon graminifolius.

## 2. Objectives

The aim of the present study was to determine acute toxicity and protective effect of HeTG against ethanol induced gastric ulcer in male rats.

## 3. Materials and Methods

### 3.1. Animals

In this study, Wistar male rats with 190 - 230 gr weight were used. Animals were kept under standard laboratory conditions (23 ± 2°C, 12 light and 12 dark cycles, and standard humidity) and had free access to water and food. The ethic committees for animal study accepted the protocol of the present study. All experiments were performed in the morning.

### 3.2. Plant

TG was collected in April from west of Iran (Kermanshah province) and authenticated by Dr. F. Attar (Department of Biology, Faculty of Sciences, University of Tehran), and a voucher specimen (No.43603) deposited in the central herbarium of Tehran University. Arial parts were dried in shadow and room temperature.

### 3.3. Extract Preparation

Plant aerial parts were powdered and 100 gr of powder was minced in 400 ml 70% ethanol. After 48 hours, the extract was filtered and speared on a flat surface. After ethanol evaporation, the extract was trimmed and weighted.

### 3.4. Drug and Chemicals

Ethanol (Merck, Germany) was used for the induction of gastric ulcer, and chloroform (Merck, Germany) was used for anesthetizing the animals. Omeprazole (Cipla, India) was used as the standard protection of gastric ulcer.

### 3.5. Acute Toxicity of TG

Twenty albino male mice were divided into four groups of five animals each. HeTG at single dose of 250, 500, 1000, and 2000 mg/Kg body weight was administered to the animals of each group intraperitoneally. Animals were observed for 48 hours after HeTG administration for mortality, clinical, and physical signs of toxicity (restlessness, dullness, agitation) (13).

### 3.6. Experiment Design

Thirty five Wistar male rats were divided into 5 groups (n = 7) including:

Group 1 (control): received distilled water (DW) (1 cc/kg)

Group 2 (control positive): received Omeprazole (OMP, 10 mg/kg) (14) 

Group 3 (extract): received HeTG extract (50 mg/kg)

Group 4 (extract): received HeTG extract (100 mg/kg)

Group 5 (extract): received HeTG extract (150 mg/kg)

The drug and extracts were given orally for 15 consecutive days (15).

### 3.7. Ethanol Induced Gastric Ulcer

Animals had starvation for forty eight hours before ulcer induction and received DW with 1% sucrose. At day fifteen, one hour after daily regimen of DW, OMP, and extracts, animals received pure ethanol (1 ml/200gr body weight) by gavage (14).

### 3.8. Macroscopic Survey

Animals were anesthetized by chloroform one hour after ethanol administration and were dissected. Their stomach was isolated and cut along greater curvature. The stomach was cleaned and speared on a flat surface (16).

### 3.9. Measurement of Ulcer Index and Calculation of Protection Rate

Gastric ulcers were measured along longitudinal axis by geritacollis under stereomicroscope and expressed in centimeter. Each five petechial lesions were counted and considered as 1 mm of ulcer. All ulcers were summed and considered as ulcer index for each animal. Protection rate was calculated by the following formula (17).

Protection rate (%)=(Control mean ulcer index – test mean ulcer index)/(Control mean ulcer index)×100

### 3.10. Microscopic Survey

For microscopic survey, gastric tissues were fixed in 10% formaldehyde and after processing and sectioning, microscopic slides were stained by Hematoxylin–Eosin methods. Tissue changes consisted of epithelial tissue and glands arrangement, edema, congestion, necrosis, hemorrhage, and leucocytes infiltration were evaluated (2).

### 3.11. Data Analysis

Data expressed as mean ± SD and were analyzed by one way ANOVA and Tukey test. P < 0.05 was considered significant.

## 4. Results

### 4.1. Acute Toxicity of TG

There was no animal mortality following single dose of intraperitoneal injection of 250, 500, 1000, and 2000 mg/kg TG extract concentrations. During observation period (48 hours), the animals did not display any clinical sign of toxicity.

### 4.2. Effect of HeTG on Ethanol Induced Gastric ulcer

**Figure 1.**
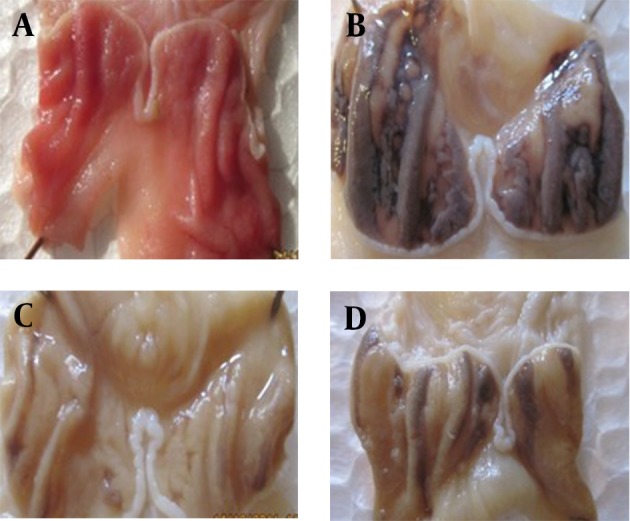
Macroscopic Photo of Dissected Stomach (a) Normal, (b) ethanol induced ulcer, (c) 50 mg/kg HeTG + Ethanol (d) 150 mg/kg HeTG + Ethanol

**Figure 2.**
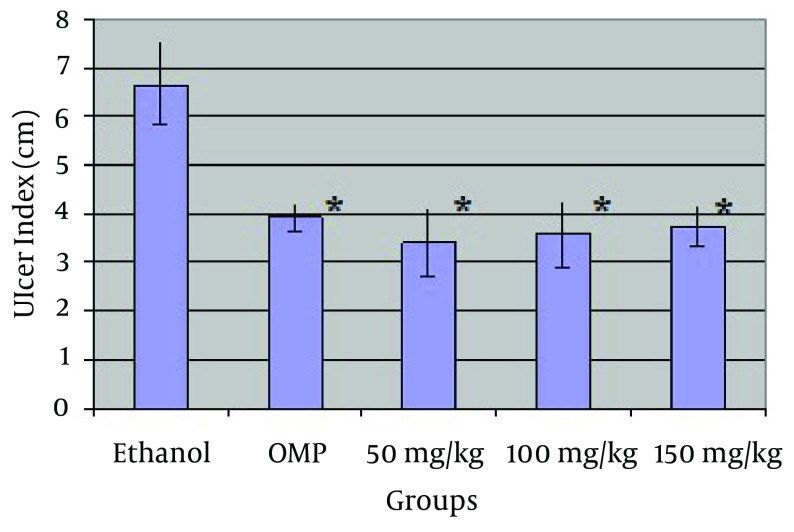
Ulcer index in HeTG and control groups

Oral gavage of ethanol induced longitudinal gastric ulcer in the glandular part of stomach of control group (Figure 1 (b)). Ulcer index in all HeTG receiving groups (50, 100, 150 mg/kg) were lower than ethanol and Omeprazole (OMP) groups significantly (P = 0.04) (Table 1 and Figure 2). HeTG (50 mg/kg) had the lowest ulcer index (3.40 ± 0.69). Difference between HeTG and OMP groups was not significant. Protection rates which indicate the protective effect of HeTG against ulcer induction in all three HeTG doses were higher than those of OMP and ethanol groups. Also the highest protection rate (%48.94) belonged to 50 mg/kg group (Table 1). 

**Table 1.** Protective Effect of HeTG on Ethanol Induced Gastric Ulcer in Rat. Ulcer Index and Protection Rate of Control and HeTG Groups Were Presented.

Groups	Ethanol	OMP	HeTG 50 mg/Kg	HeTG 100 mg/Kg	HeTG 150 mg/Kg
**Ulcer index (cm) ^a^**	6.66 ± 0.85	3.94 ± 0.28^b^	3.40 ± 0/69^b^	3.57 ± 0.67^b^	3.73 ± 0.42^b^
**Protection rate (%)**	-	40.84%	48.94%	46.39%	43.99%

^a^ Values express as means ± standard deviation. The mean reference is significant at 0.05 level (n = 7)

^b^ P < 0.05 compared with respective ethanol control group

### 4.3. Microscopic Evaluation

**Figure 3.**
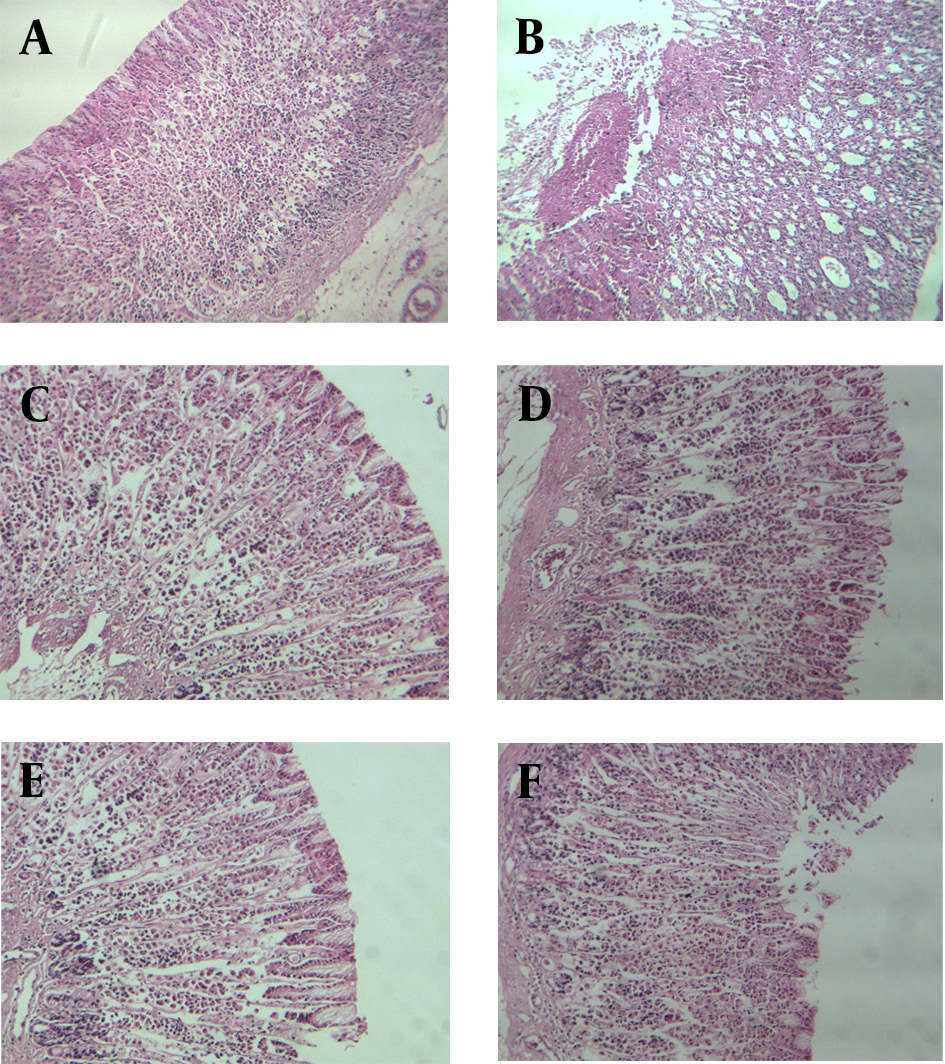
Microscopic Photos of Gastric Tissue, Stained with Hematoxylin and Eosin Methods (×100) (a) normal, (b) ethanol, (c) HeTG (50 mg/kg) + Ethanol, (d) HeTG (100 mg/kg) + Ethanol, (e) HeTG (150 mg/kg) + Ethanol, (f) OMP (10 mg/kg) + Ethanol

Microscopic evaluation of ethanol induced gastric lesion showed severe bleeding, leucocytes and RBC infiltration, epithelial and glandular destruction, and cellular irregularity. The ulcers were limited to gastric mucosa and there were no submucosal lesions (Figure 3 (b)). HeTG decreased ethanol induced necrosis and tissue damage. In HeTG groups (especially 50 mg/kg), cellular arrangement and vascular structure of lamina propria were relatively normal, but cellular density was less than normal status (Figure 3 (c)).
